# SOCIOECONOMIC STATUS AND COGNITIVE DIFFICULTY: DIMINISHED RETURNS AMONG OLDER BHUTANESE REFUGEES IN THE US

**DOI:** 10.1093/geroni/igae098.0769

**Published:** 2024-12-31

**Authors:** Katherine Kitchens, Jaclyn Kirsch

**Affiliations:** The University of Texas Arlington, Arlington, Texas, United States; The University of Texas Arlington, Arlington, Texas, United States

## Abstract

Refugee populations report cognitive difficulties at a rate significantly higher than observed among older immigrants and native-born U.S. adults. There is evidence that socioeconomic status acts as a buffer against mild cognitive impairment. This study aimed to examine how socioeconomic status is differentially associated with cognitive difficulties between older Bhutanese refugees and other refugee groups in the United States (N = 5,666), using the 2021 American Community Survey five-year estimates and the official refugee counts from the Department of Homeland Security. Binary logistic regression analyses examined the moderating effect of Bhutanese refugee status on the association of income level and high school completion with self-reported cognitive difficulty, controlling for demographic and integration variables. High school completion was associated with a 43% decrease in the odds of cognitive difficulty among older refugees (AOR = 0.57, p<.001, 95% CI [0.43,0.75]), yet educational attainment was not a significant protective factor for older Bhutanese refugees, who had almost three times the odds of experiencing a cognitive difficulty compared to other refugee groups (AOR = 2.86, p<.001, 95% CI [1.86,4.40]). Interactions with income level indicated that living above 138% of the federal poverty level significantly increased the odds of reporting a cognitive difficulty (AOR = 2.86, p<.001 95% CI [1.86,4.40], suggesting that higher income does not serve as a protective factor against cognitive difficulty for older refugees from Bhutan, giving evidence of diminished returns. Findings suggest a need for policies that enable Bhutanese refugees to convert their resources more effectively into concrete cognitive health outcomes.

